# Temporal Trends in Sports Participation among Adolescents between 2001 and 2015: A French School- and Territory-Based Study

**DOI:** 10.3390/ijerph15071335

**Published:** 2018-06-26

**Authors:** Maxime Luiggi, Maxime Travert, Jean Griffet

**Affiliations:** Institute of Movement Sciences, Aix Marseille University, CNRS, 13009 Marseille, France; maxime.travert@univ-amu.fr (M.T.); jean.griffet@univ-amu.fr (J.G.)

**Keywords:** public health, exercise, sports, health policy, Western Europe, health promotion

## Abstract

Improving adolescents’ levels of sport and physical activity (PA) is an official public health issue. French national government plans were launched in 2001, 2006, and 2011 to improve the participation levels of citizens. These plans should be monitored. To date, information on temporal trends in sports has come from the national population. However, no data are available to measure temporal trends in different territories across the country. Our study aimed to measure these trends among a representative sample of adolescent students of the third biggest French region (Bouches-du-Rhône), but also one of the poorest, between 2001 and 2015. Three surveys were conducted in 2001, 2008, and 2015 in high schools (*n* = 3218). Logistic regressions adjusted for age were used to determine the impact of socioeconomic status (SES) on sports participation and to measure the changes in sport participation rates. Participation declined among all subgroups of adolescents: from 79.0% to 65.8%. The greatest decrease was observed for boys with a high SES, whilst the lowest was for the high-SES girls. We observed that SES inequalities in access to sport increased among the girls, whilst they reduced among the boys. National government plans seem to have had limited success in this territory. Next to national studies, there is a need to develop territory specific studies which could show important disparities across the national territory.

## 1. Introduction

Many studies show the positive effect of physical activity (PA) on adolescents’ health [1,2] and weight status [3,4]. Sports help to attain daily recommended levels of PA [5] and have an impact on psychological and social health [6]. Promoting sports participation among citizens and supporting local sports organizations are recommended by the World Health Organization [7]. Since the Lisbon Treaty (2009) came into force, the aim in the European Union has been to “promote sport and physical activity at the policy level”. Moreover, PA and sports participation during adolescence are predictors of PA and sport participation in adulthood [8,9,10], where a physically active lifestyle helps to prevent cancer, type II diabetes, osteoporosis [11], and cardiovascular diseases [12,13]. Adherence to sports by the largest possible number of adolescents thus constitutes, in the short and long term, a major public health issue.

This importance has been highlighted in multiple plans developed by the French Government during the last decades to improve physical and sports activities at all ages of life [14]. In 2001, 2006 and 2011, national plans called first “MangerBouger” (EatMove) then “Plan National Nutrition Santé” (National Healthy Nutrition Plan) (PNNS), aimed to “reduce, through specific actions, social health inequalities in the field of nutrition within general prevention actions” and “develop physical and sports activity and limit sedentary living”. To achieve these objectives many initiatives were launched across the national territory such as (i) communication measures for informing the public and health professionals of the benefits of physical activity, (ii) actions for promoting and developing physical and sports activities in populations with disabilities, in disadvantaged situations, and of elderly or chronically ill persons [14] (pp. 28–30). Monitoring sport participation is one of the essential components to evaluate these governmental plans [15]. It could provide important evidence to inform public health officials and to identify the least active populations [16]. To date, information on temporal trends in sports participation in France has come from the national population [17,18,19,20]. In the Esteban report, a stable level of organized sport participation was observed among adolescents aged 15–17 [18]. However, this study did not measure the sport participation rates in an unorganized context. Thus, we cannot draw a conclusion regarding the evolution of the total sport phenomenon (organized and unorganized). In the Eurobarometer reports, the level of stability in French adolescent sport participation (aged 15–24), both in unorganized and organized context, was also observed between 2004 and 2015. In 2004, 64% of adolescents declared to play sport one or two times a week [19]. They were 66% in 2015 [20].

Along with these national findings, it is also important to estimate levels of participation in different territories across the country. It has been shown that factors influencing sport participation differed according to the area of residence. For example, the effects of socioeconomic status (SES) and sex were different between countries and areas within countries [21]. Toftegaard-Støckel et al. showed that in some Danish localities, the influence of low SES differed by sex whilst in others it remained the same [22]. Finally, environmental factors such as ease of access to sports infrastructures or local opportunities [23,24,25] could differ across the national territory. It is likely that local particularities could lead to different levels of participation compared to the national level. Such findings could provide important evidence to help the development of future sport promotion programs. However, in the Eurobarometer, the French sample size was too low to estimate levels of participation in particular subgroups of adolescents living in different areas. Each year of the Eurobarometer surveys, the sample size was 1000 [19,20]. More specifically, in 2004, the 15–24 age range group included 168 participants. In 2015, it included 154 participants. Considering France has 96 administrative divisions (called departments), that puts the average participant per department at less than two participants aged 15–24. Such a sample size is too small to estimate levels of participation in each French department, let alone to estimate participation by sex and SES of adolescents.

The main purpose of this study was to measure the temporal trends in adolescents’ sports participation in a specific French territory between 2001 and 2015. The third biggest department of France (Bouches-du-Rhône) [26] represents an interesting study case. This is mainly an urban area, but one which also provides important natural space such as seacoast and mountain. More specifically, this department is one of the poorest of France [27], with a large proportion of high schools subject to a national educational plan to improve pupils’ success (see priority education network/area indicators at: https://www.reseau-canope.fr/education-prioritaire/comprendre/donnees-cles.html]. It is recognized that these territories were also those with the fewest sports facilities per inhabitant [28]. Given the essential role of the accessibility of sports infrastructures to understanding participation, one could argue that temporal trends in this area would be different than those observed at the national level. Results would provide important information to the development of future sport promotion programs. Three assessment points were chosen with 7-year intervals. Sociodemographic factors (sex, age, socioeconomic status) were investigated in relation with sport participation.

## 2. Materials and Methods

### 2.1. Participants and Procedure

Three sets of data collection on French adolescent students’ health were carried out between 23 March and 13 April 2001; 1 February and 1 April 2008; and 2 February and 10 April 2015, in 11 junior and 19 senior high schools in Bouches-du-Rhône, Provence, France. Each survey (2001, 2008, and 2015) was approved by the Rector of the school academy—also named Chancellor of the Universities—where our surveys were conducted. He is the territorial represent of the Minister of National Education and Research. In that respect, he has the authority concerning initiatives or research launched among schools (from primary to university levels).

#### 2.1.1. Sampling Design

In this area, there were 136 public junior high schools of which 23 were classified as priority education networks (REP) [29]. Among 80 senior high schools, 30 were classified as experimental school success schemes (DERS), suburban hope neighborhood institutions (EQEB) or priority education areas (ZEP). These schools are characterized with a low level of pupil achievement and a greater proportion of parents with low socioeconomic status, and are often located in sensitive urban zone (ZUS) (see [30,31] for priority education networks scoreboard in 2008 and 2015). Fourteen key initiatives were launched by public health officials towards these schools. Briefly, among them, to encourage fewer students per class, to spend more time in class per week, and to give support for homework outside of official school hours (for a complete list, see http://www.education.gouv.fr/cid76427/refonder-education-prioritaire.html). To keep our sample representative of the students in the department of Bouches-du-Rhône, we selected high schools so as to maintain the proportion of “classic” establishments and those classified under REP, DERS, EQEB, or ZEP. Each year, we randomly selected among junior high schools 3 REP and 16 “classic” establishments. Among the senior high schools, we randomly selected 4 DERS, EQEB, or ZEP, and 7 “classic” establishments. The adolescents invited to fill out the surveys were in their third year of junior high school or their last year of senior high school (9th and 12th grade). Only adolescents who agreed to participate and who returned signed parental informed consent forms were included in the study (*n* = 3218; response rates: 2001: 99.3%; 2008: 98.4%; 2015: 98.5%). Every student participated in the survey under the supervision of a physical education teacher and a researcher.

#### 2.1.2. Study Sample

In 2001, the sample comprised 878 adolescents (50.8% girls, Age = 16.6, SD = 1.5), representing 2.8% of the students in these grades in Bouches-du-Rhône. In 2008, the sample comprised 1321 adolescents (60.8% girls, Age = 16.2, SD = 1.88), representing 3.4% of the students in these grades in Bouches-du-Rhône. In 2015, the sample comprised 1019 adolescents (56.4% girls, Age = 16.3, SD = 1.56), representing 3.0% of the students in these grades in Bouches-du-Rhône.

### 2.2. Instruments/Measures

The variables covered in the questionnaire concerned demographics and sports participation.

#### 2.2.1. Demographics

Students were asked to report their age, sex, and the head of their family’s employment status. Similarly to other French studies, the head of their family’s employment status was used to estimate the SES. Adolescents had to answer the question: ‘What is the occupation of the head of your family?’ by checking the applicable box (farmer; merchant, artisan, company director; intellectual occupations (engineer, doctor/physician, et cetera); intermediate professions (teacher, nurse, technician, et cetera); salaried employee; manual worker; retiree; unemployed; other). In view of previous studies on the link between SES and sport participation, with some focusing on high and low SES for the analyses [21,22], we also chose to divide SES into two groups (high and low). The division into two groups was also relevant to obtain the most adequate sample size in each subgroup of adolescents (sex x SES). Ideally, the sample size by subgroups would be around 350 to estimate precisely the sport participation rates in the parent population. To calculate it, we used the formula $n=\left[z^{2}\;*\;P\;*\;\left(1-P\right)\right]\;/\;d^{2}$, where *n* is sample size needed, *z* is the statistic for the level of confidence (0.05 chosen, i.e., 1.96), *P* the prevalence expected (0.65 as national findings), and *d* the allowable error (0.05 chosen). Given that our sample size was between 878 and 1321, it was more reliable to divide SES into two groups rather than three or more (878/4 = 220; 878/6 = 146). It helped obtain the most precise estimation of sport participation rates in the parent population.

For the cutting point, our criterion was average annual income (AAI). In 2013, in the department of Bouches-du-Rhône, AAI was 21,955 € per inhabitant [32,33]. Occupations which had an AAI less than 21,955 € were considered as having low socio-economic status. These were the salaried employees (AAI = 15,368 €), manual workers (AAI = 17,335 €), retirees (AAI = 20,590 €), or unemployed persons (14,050 €) (*n* = 1445). Occupations which had an AAI over 21,955 € were considered as having high socio-economic status. These were persons in intellectual occupations (AAI = 39,623 €) or intermediate occupations (AAI = 24,536 €) (*n* = 1503). Young people whose parents’ occupations were unknown were excluded from the SES analyses (*n*_2001_ = 134, *n*_2008_ = 63, *n*_2015_ = 73). Official statistics did not provide information about the AAI of farmers. Thus, they were classified as unknown (*n*_2001_ = 22, *n*_2008_ = 22, *n*_2015_ = 8). In 2001, 44.2% of adolescents were from low-SES families, 40.5% from high SES, and 15.3% have an unknown SES. In 2008, 46.4% of adolescents were from low-SES families, 48.8% of high SES, and 4.8% of unknown SES. In 2015, 43.6% of adolescents were from low-SES families, 49.3% of high SES, and 7.2% of unknown SES.

#### 2.2.2. Sport Participation

The outcome variable was sport player, coded as ‘yes’ or ‘no’. Sport was defined according to the definition of the Council of Europe [34]: “Sports means all forms of physical activity which, through causal or organized participation, aim at expressing or improving physical fitness and mental wellbeing, forming social relationships or obtaining results in competition at all levels.” As questioned by Lamprecht et al., students were invited to report their sport participation outside of physical education classes at school (“Apart from physical education classes at school, do you practice sports?”) [35]. Students who said ‘yes’ were asked to report the sport they prefer to practice outside school, their preferred context of participation (organized or unorganized), and their usual amount of practice per week (for both organized and unorganized participation). Preferred activities were listed in a separate table, and a committee of physical education teachers and researchers in sport sciences (*n* = 6) was formed to decide whether or not these activities could be considered sports with regard to the definition of the Council of Europe [34]. Students playing their preferred activity—considered a sport by the committee—and practicing it for 1 h or more per week (regardless of the context of participation) were classified as sport players. Students who declared an activity which was not considered a sport by the committee were classified non-sport players in the analyses (*n* = 5). The lists of preferred sport activities considered as being sports and as non-sports by the committee are presented in Supplementary Materials Table S1 and S2, respectively.

For the reader’s interest, typically, in France, organized sport is played in community clubs. At the national level, there was around 38% of 15–24 year olds holding a federation license in 2016 (see http://www.injep.fr/article/tableaux-statistiques-relatifs-au-recensement-des-licences-sportives-de-2016-11533.html). This rate was around 21% for organized sport at school, according to official statistics of the National Union of School Sport (see https://unss.org/wp-content/uploads/2018/02/UNSS-PDNSS-2016-2020.pdf).

### 2.3. Data Analysis

Statistical analyses were performed using SPSS 18.0.0 (SPSS Inc., Chicago, USA). Firstly, sport participation rates were calculated by sex, SES (low/high), and year. The chi-square statistical test was used to give general effects of sex and SES on sport participation. Secondly, a binary logistic regression adjusted for age was performed to determine the effect of SES by year and by sex. Thirdly, multiple binary logistic regressions adjusted for age were performed to determine the changes in sport participation between 2001 and 2008, between 2008 and 2015, and between 2001 and 2015 by sex and SES. No specific analyses were performed to measure the temporal trends in preferred sports activities chosen by adolescents between 2001 and 2015. This analysis will be done in subsequent papers treating that topic specifically.

## 3. Results

Sports players were 79.0% in 2001, 71.8% in 2008, and 65.8% in 2015. A greater proportion preferred the organized context of participation (2001: 57.2%; 2008: 57.5%; 2015: 61.0%). Table 1 provides sociodemographic characteristics and sport participation rates by sex, SES, and year.

### 3.1. General Effects of Sex and SES by Year on Sport Participation

In 2001, 2008, and 2015, sport participation was higher in adolescent boys compared to adolescent girls (diff = +18.4%, Chi^2^ = 45.20; diff = 22.3%, Chi^2^ = 77.38, respectively; diff = 21.3%, Chi^2^ = 68.18, overall: *p* < 0.001).

In 2008 and 2015, sport participation was higher in adolescents of a high SES compared to low SES (diff = +14.0%, Chi^2^ = 30.57, *p* < 0.001; diff = +7.7%, Chi^2^ = 6.30, *p* < 0.05, respectively)

In 2001, 2008, and 2015, sport participation was lower in adolescent girls with a low SES compared to adolescent boys with a low SES (diff = −19.5%, Chi^2^ = 21.28; diff = −26.2%, Chi^2^ = 40.46; diff = −27.7%, Chi^2^ = 45.70, respectively; overall *p* < 0.001).

In 2001, 2008, and 2015, sport participation was lower in adolescent girls with a high SES compared to adolescent boys with a high SES (diff = −18.5%, Chi^2^ = 20.50; diff = −15.5%, Chi^2^ = 23.05; diff = −15.5%, Chi^2^ = 19.18, respectively; overall *p* < 0.001).

### 3.2. Effects of SES on Sport Participation by Sex and Year

Table 2 presents the results of binary logistic regressions adjusted for age by year and by sex performed to see the effect of SES on adherence to sport participation.

In 2001, 2008, and 2015, there was no SES effect on the boys’ sports participation.

In 2001, there was no SES effect on the girls’ sports participation.

In 2008 and 2015, there was an SES effect on the girls’ sports participation: girls with a low SES were less likely than girls with a high SES to play sport—OR: 0.54 (95% CI: 0.40–0.73) and OR: 0.61 (95% CI: 0.42–0.87), respectively.

### 3.3. Evolution of Sports Practice between 2001 and 2008, 2008 and 2015, 2001 and 2015 by Sex and SES

Table 3 presents the results of binary logistic regressions adjusted for age by sex and SES performed to measure trends in sport participation.

In 2008, girls with low SES were less likely to play sport compared to 2001.

In 2015, girls and boys with a high SES were less likely to play sport than in 2008.

In 2015, compared with 2001, all the adolescent populations were less likely to play sport.

**Figure 1:**
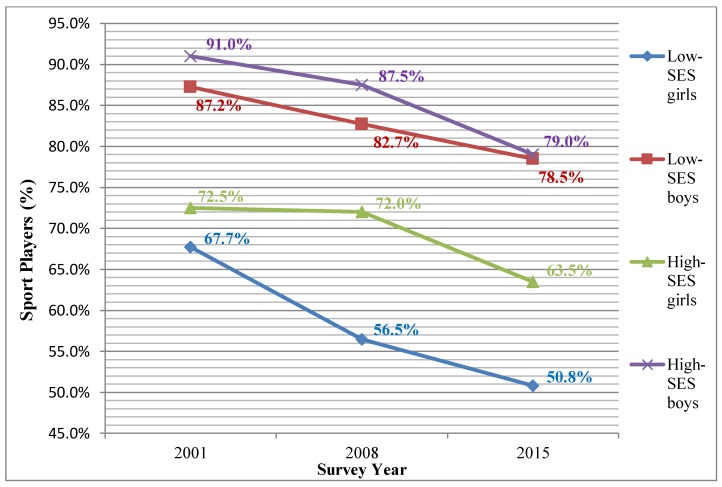
Sport participation rates by sex and socioeconomic status (SES) by year.

Figure 1 gives a visual overview of temporal trends in sport participation by sex and SES.

## 4. Discussion

Our study treated both the effects of SES by sex and year and the evolution in sports participation by sex and SES from 2001 to 2015.

As regards the SES effects, consistent with previous findings, we observed that, regardless of SES, girls practice sports less than boys [5,8,21,22] and that a low SES is a barrier to sports practice [21,22]. In addition, we observed no differences in sport participation between low-SES boys and high-SES boys. Contrarily, we observed a difference in sport participation between low-SES girls and high-SES girls in 2008 and 2015.

Regarding the temporal trends in sports, we observed a decline in adolescent sport participation, regardless sex or SES. This variation in sport participation was different from that seen in the Eurobarometers [19,20], which showed stability in participation between 2004 and 2015 among adolescents aged 15–24. The lower rate locally observed could be partially explained by the large proportions of families with a low SES [27] and the low availability of sports infrastructures [28] in this area. It is well known that these factors influence adolescent non-sport participation [21,22,23,24,25]. In addition, the results showed different magnitudes in decreases by sex and SES. Adjusted for age, the results showed that boys with a high SES were the most concerned by the drop in sport participation whilst the girls with a high SES least concerned. These different magnitudes in decreases for the adolescents with a high SES led to different trends in social inequalities in access to sport by sex. The differences in sport participation rates between boys with a low SES and those with a high SES diminished over time. In 2001 and 2008, a slight difference was observed, whilst in 2015, the amount of boys playing sports with a low SES was of the same proportion as those with a high SES playing sports. On the contrary, there were increasing inequalities linked to SES in access to sport among the girls. In 2001, a difference existed between high-SES and low-SES girls but it was not statistically significant. However, from 2008 to 2015, the difference in sport participation rates increased and became significant. To our knowledge, such results have never been observed in the European Union, nor in France. Further studies should be conducted in other areas of France and European countries to observe whether these trends are observable elsewhere. These studies could include factors known to be linked to girls’ non-participation, such as access constraints or perceived safety around the sport facilities [36,37,38]. One could argue that these factors became more influential over the time among the girls with a low SES compared with those with a high SES. Future studies could test this hypothesis in different countries and areas within countries. In terms of public health promotion, results would provide important evidence to develop adequate sport and physical activities promotion initiatives.

Along with the French national trends observed in the Eurobarometers, these results highlighted the need to develop territory specific studies within the country. As we mentioned in the introduction, the former reflected the global situation without being able to provide estimation of participation in specific territories. The sample sizes were too low. Our study revealed the importance of this problem. In spite of the stability in national adolescent sport participation, the department of Bouches-du-Rhône presented an important decline. In addition, this revealed, for the first time, important disparities in participation across the year. This is an important issue to help understand why inequalities increased among the girls, whilst they reduced among the boys. Future researches should be done. In terms of public policy, it is important to consider local specificities and their influence on sport participation, to develop the most adequate sport and physical activities promotions programs. To improve local knowledge, firstly, it would be interesting to conduct similar studies in both (i) the poorest, (ii) the richest areas of France. It is likely that temporal trends in sports will be quite different as a function of the overall SES levels and sports infrastructures of each territory. Such findings could provide important information to help the development of future sport and PA promotions programs based on scientific evidence.

### Study Strengths and Limitations

One of the major features of our study was the three-assessment points during a 15-year period within the same methodology in a specific French territory. This is the first time that a territory-specific study has been conducted since the launch of the physical and sports activities promotions programs by the French Government. In addition, it was considered that having invited students’ populations in junior and senior high schools to participate was of great importance. With this methodology, we were able to select high schools in order to keep the sample representative in terms of level of pupil achievement, parents’ occupations, and place of residence. In our opinion, the relative important representativeness of the sample ensures reliable results. They should represent valuable information for public health officials.

This study also entailed several limitations.

Firstly, and regrettably, our study could not help to determine distinctly the sport participation rates in organized and unorganized context. Adolescents were asked to report how many hours per week they played sport, regardless of the context of participation. However, information related to the times spent in each context was not provided. Adolescents were asked to report their preferred context of participation. Although we observed stability in the preferred context of participation (organized), we cannot assume that their preferred context of participation is equal with their usual and more frequent context of sport participation. It is thus impossible to address whether the decrease in sport participation observed was more pronounced in the unorganized or the organized context. Future studies could consider all these methodological questions in order to obtain the most wide and precise information regarding the temporal trends in adolescent sport participation.

Secondly, public health policies aim to improve PA, and sports are viewed as a means to attain recommended levels of PA. In this study, we measured only sport participation rates. Although we observed a decrease in sport participation, results did not provide information about the temporal trends in overall levels of PA, for example, active transportation. Future studies should combine measures of sport participation and levels of PA as proposed in the Global Physical Activity Questionnaire [39], which showed acceptable reliability [40].

Thirdly, the seven-year interval between surveys is too large to accurately situate the variations in sports practice rates. It is thus impossible to connect them to precisely situated environmental factors and actions.

Finally, future studies on trends in youth sports practice should include more factors [41] that are likely to provide explanations. For example, sociocultural factors favoring adherence to sports, such as parents’ sports practice [22] or family structure [42], were not used in our survey protocols. Taking these factors into account could help provide a better understanding of young people’s loss of interest in sports.

## 5. Conclusions

Sports participation rates (organized and unorganized) of adolescent students living in this area were lower in 2015 compared to 2001, regardless of sex and SES. The results are in contrast with those observed by national reports. These findings suggest a limited effect of the French national plans to improve the sport activities levels of adolescents living in the department of Bouches-du-Rhône. The specificity of this area could explain these differences. Notably, the results highlighted that social inequalities in sport participation changed differently across the adolescents’ sex. The differences in sport participation rates between boys with a low SES and those with a high SES diminished over the time. On the contrary, there were increasing inequalities linked to SES in access to sport among the girls. Future researches should be done to understand this phenomenon. Alongside national studies, there is a need to develop territory-specific studies which could show important local phenomenon and disparities across the national territory.

## Figures and Tables

**Table 1:** Sociodemographic characteristics and sport participation (%) by sex, socioeconomic status (SES), and year.

*Variables*		2001			2008			2015		
		*n*	Sport Player (%)	*p*-Value	*n*	Sport Player (%)	*p*-Value	*n*	Sport Player (%)	*p*-Value
*Sex*										
	Girls	446	70.0%		803	63.0%		548	56.6%	
	Boys	432	88.4%	<0.001	518	85.3%	<0.001	462	77.9%	<0.001
*SES*										
	Low SES	388	77.6%		613	64.9%		444	62.6%	
	High SES	356	81.7%	0.16	645	78.9%	<0.001	502	70.3%	<0.05
	Unknown	134	76.1%		63	65.1%		73	53.4%	
*Sex* × *SES*										
	Low-SES girls	192	67.7%		416	56.5%		238	50.8%	
	Low-SES boys	196	87.2%	<0.001	197	82.7%	<0.001	200	78.5%	<0.001
	High-SES girls	178	72.5%		357	72.0%		271	63.5%	
	High-SES boys	178	91.0%	<0.001	288	87.5%	<0.001	229	79.0%	<0.001
*All*		878	79.0%		1321	71.8%		1019	65.8%	

Note. Sport player is defined as an adolescent who declared playing sport at least one hour per week. Sex × SES: Groups creation based on sex (Girls/Boys) and SES (Low/High) of adolescents.

**Table 2:** Odds ratio (OR) and 95% confidence interval (CI) describing the relationship between sport participation and socioeconomic status by sex and year, adjusted for age.

*Variables*		2001				2008				2015			
		Boys		Girls		Boys		Girls		Boys		Girls	
		OR	95% CI	OR	95% CI	OR	95% CI	OR	95% CI	OR	95% CI	OR	95% CI
	Age	1.08	[0.89; 1.31]	1.02	[0.89; 1.16]	0.83 **	[0.72; 0.94]	0.90 **	[0.83; 0.97]	0.89	[0.78; 1.03]	1.02	[0.92; 1.14]
Socioeconomic status													
	High	1		1		1		1		1		1	
	Low	0.67	[0.35; 1.31]	0.81	[0.52; 1.27]	0.71	[0.42; 1.19]	0.54 ***	[0.40; 0.73]	0.98	[0.61; 1.56]	0.61 **	[0.42; 0.87]
		*R*² = 0.01		*R*² = 0.004		*R*² = 0.04		*R*² = 0.05		*R*² = 0.01		*R*² = 0.02	

No interaction was found between age and socioeconomic status in relation to sports participation. ** *p* < 0.01; *** *p* < 0.001. *R*²: Nagelkerke *R*-square.

**Table 3:** Odds ratio (OR) and 95% confidence interval (CI) describing the relationship between sport participation and year by sex and SES, adjusted for age.

Survey Year		Girls with Low SES		Girls with High SES		Boys with Low SES		Boys with High SES	
		OR	95% CI	OR	95% CI	OR	95% CI	OR	95% CI
2008 ^a^		0.61 **	[0.43; 0.87]	0.92	[0.61; 1.38]	0.64	[0.36; 1.13]	0.66	[0.35; 1.25]
	*R*²	0.02		0.01		0.02		0.01	
2015 ^b^		0.78	[0.57; 1.08]	0.70 *	[0.50; 0.98]	0.81	[0.49; 1.35]	0.56 *	[0.34; 0.90]
	*R*²	0.01		0.02		0.05		0.03	
2015 ^a^		0.49 ***	[0.33; 0.74]	0.66 *	[0.44; 1.00]	0.51 *	[0.30; 0.88]	0.38 **	[0.21; 0.69]
	*R*²	0.04		0.01		0.03		0.05	

^a^ reference category: year 2001; ^b^ reference category: year 2008; * *p* < 0.05; ** *p* < 0.01; *** *p* < 0.001. *R*²: Nagelkerke R-square.

## Supplementary Materials

The following are available online at http://www.mdpi.com/1660-4601/15/7/1335/s1, Table S1: List of sport activities practiced by adolescents by year and considered as sport according to council of Europe definition, Table S2: List of sport activities practiced by adolescents by year and no considered as sport according to Council of Europe definition.
