# Supplementary material for: Temporal Trends in Sports Participation among Adolescents between 2001 and 2015: A French School- and Territory-Based Study
Source: Int J Environ Res Public Health. 2018 Jun 26;15(7):1335. doi: 10.3390/ijerph15071335 (PMC6068882; doi:10.3390/ijerph15071335)
Supplement: Supplementary file 1 [file ijerph-15-01335-s001.pdf]

Supplementary Materials: Temporal Trends in Sports Participation among Adolescents between 2001 and 2015: A French School- and Territory-Based Study

Maxime Luiggi *, Maxime Travert and Jean Griffet

Table S1. List of sport activities practiced by adolescents by year and considered as sport according to council of Europe definition.

Year	2001		2008		2015	
**SPORT**	**n**	**%**	**n**	**%**	**n**	**%**
AEROBIC	0	0.0%	0	0.0%	2	0.3%
AIKIDO	5	0.7%	0	0.0%	3	0.4%
AIRSOFT	2	0.3%	0	0.0%	1	0.1%
AMERICAN FOOTBALL	0	0.0%	1	0.1%	2	0.3%
ARCHERY	0	0.0%	1	0.1%	0	0.0%
ARTISTIC GYMNASTIC	0	0.0%	0	0.0%	4	0.6%
ATHLETICS	14	2.0%	8	0.8%	9	1.3%
BADMINTON	13	1.9%	13	1.4%	23	3.4%
BALLET DANCE	0	0.0%	0	0.0%	5	0.7%
BASEBALL	0	0.0%	1	0.1%	0	0.0%
BASKET-BALL	40	5.8%	42	4.4%	38	5.7%
BIKING	0	0.0%	34	3.6%	4	0.6%
BMX	2	0.3%	0	0.0%	4	0.6%
BMX BICROSS	0	0.0%	0	0.0%	1	0.1%
BODYBOARD	2	0.3%	1	0.1%	0	0.0%
BODYBUILDING	12	1.7%	21	2.2%	26	3.9%
BOXING	25	3.6%	12	1.3%	30	4.5%
BULLFIGHTING	0	0.0%	2	0.2%	0	0.0%
CANYONING	0	0.0%	1	0.1%	0	0.0%
CAPOEIRA	0	0.0%	2	0.2%	0	0.0%
CLIMBING	19	2.7%	23	2.4%	8	1.2%
CROSS-COUNTRY CYCLING	1	0.1%	1	0.1%	2	0.3%
DANCING	75	10.8%	102	10.8%	105	15.7%
FENCING	0	0.0%	2	0.2%	2	0.3%
FIGURE SKATING	0	0.0%	0	0.0%	1	0.1%
FITNESS	10	1.4%	14	1.5%	12	1.8%
FLAG FOOTBALL	1	0.1%	1	0.1%	0	0.0%
FREE-DIVING	1	0.1%	1	0.1%	0	0.0%
FRENCH BOXING	3	0.4%	0	0.0%	6	0.9%
GOLF	0	0.0%	2	0.2%	1	0.1%
GYMNASTIC	9	1.3%	7	0.7%	9	1.3%
HANDBALL	10	1.4%	8	0.8%	9	1.3%
HIKING	0	0.0%	2	0.2%	0	0.0%
HIP-HOP DANCE	1	0.1%	0	0.0%	6	0.9%
HORSE RIDING	24	3.5%	28	3.0%	24	3.6%
ICE HOCKEY	1	0.1%	1	0.1%	3	0.4%
ICE SKATING	2	0.3%	4	0.4%	1	0.1%
JUDO	7	1.0%	4	0.4%	9	1.3%
JUJITSU	0	0.0%	0	0.0%	2	0.3%

KARATE	8	1.2%	8	0.8%	9	1.3%
KAYAK	1	0.1%	1	0.1%	1	0.1%
KENDO	0	0.0%	0	0.0%	1	0.1%
KRAV MAGA	0	0.0%	0	0.0%	1	0.1%
KUNG-FU	2	0.3%	3	0.3%	1	0.1%
LATIN DANCE	0	0.0%	0	0.0%	1	0.1%
LONGBOARDING	0	0.0%	0	0.0%	1	0.1%
MARTIAL ARTS	6	0.9%	25	2.6%	2	0.3%
MIXED-MARTIAL ARTS	0	0.0%	0	0.0%	1	0.1%
MODERN JAZZ DANCE	1	0.1%	0	0.0%	2	0.3%
MOTOCROSS RIDING	0	0.0%	0	0.0%	3	0.4%
MOTORCYCLE SPORT	1	0.1%	1	0.1%	0	0.0%
ORIENTAL DANCE	0	0.0%	0	0.0%	1	0.1%
ORIENTEERING RACE	0	0.0%	0	0.0%	2	0.3%
PADEL	1	0.1%	1	0.1%	0	0.0%
PAINTBALL	2	0.3%	1	0.1%	0	0.0%
PARKOUR	0	0.0%	0	0.0%	3	0.4%
PETANQUE (FRENCH BOWLS)	2	0.3%	1	0.1%	0	0.0%
POWER TRAINING	0	0.0%	0	0.0%	1	0.1%
QUAD	0	0.0%	1	0.1%	0	0.0%
RESCUE SWIMMING	0	0.0%	1	0.1%	1	0.1%
ROAD BYCYCLE RACING	21	3.0%	0	0.0%	2	0.3%
ROLLER HOCKEY	0	0.0%	0	0.0%	1	0.1%
ROLLER SKATING	7	1.0%	8	0.8%	1	0.1%
ROWING	6	0.9%	4	0.4%	1	0.1%
RUGBY FOOTBALL	12	1.7%	15	1.6%	22	3.3%
RUNNING	24	3.5%	93	9.8%	21	3.1%
RYTHMIC GYMNASTIC	1	0.1%	2	0.2%	2	0.3%
SAILING	0	0.0%	10	1.1%	1	0.1%
SAMBO BOXING	1	0.1%	0	0.0%	1	0.1%
SCUBA DIVING	0	0.0%	5	0.5%	0	0.0%
SELF DEFENSE	1	0.1%	1	0.1%	2	0.3%
SHOOTING SPORT	0	0.0%	0	0.0%	1	0.1%
SKATEBOARDING	9	1.3%	19	2.0%	6	0.9%
SKIING	16	2.3%	2	0.2%	6	0.9%
SNOWBOARDING	13	1.9%	1	0.1%	0	0.0%
SOCCER	134	19.3%	198	20.9%	115	17.2%
SQUASH	0	0.0%	2	0.2%	5	0.7%
SURFING	3	0.4%	3	0.3%	2	0.3%
SWIMMING	39	5.6%	47	5.0%	29	4.3%
SYNCHRONIZED SWIMMING	5	0.7%	5	0.5%	2	0.3%
TABLE TENNIS	3	0.4%	5	0.5%	2	0.3%
TAEKWONDO	4	0.6%	5	0.5%	4	0.6%
TENNIS	54	7.8%	69	7.3%	29	4.3%
THAI BOXING	1	0.1%	0	0.0%	8	1.2%
TRAMPOLINING	0	0.0%	0	0.0%	2	0.3%
TRIATHLON	0	0.0%	1	0.1%	0	0.0%
ULTIMATE FRISBEE	0	0.0%	1	0.1%	0	0.0%
VOLLEYBALL	21	3.0%	22	2.3%	13	1.9%
WALKING	0	0.0%	27	2.8%	0	0.0%
WATERPOLO	2	0.3%	3	0.3%	0	0.0%
WINDSURFING	13	1.9%	18	1.9%	3	0.4%

YOGA	0	0.0%	0	0.0%	1	0.1%
YOSEIKAN BUDO	0	0.0%	0	0.0%	1	0.1%
ZUMBA DANCE	1	0.1%	0	0.0%	4	0.6%
**All**	**694**	**100.0%**	**948**	**100.0%**	**670**	**100.0%**

Note. In 2001. 694 adolescents play sport at least one hour per week. Of them 21 (3.0%) declared that Volley-Ball is their preferred sport activity.

Table S2. List of sport activities practiced by adolescents by year and no considered as sport according to Council of Europe definition.

**Year**	**2001**		**2008**		**2015**	
**SPORT**	**n**	**%**	**n**	**%**	**n**	**%**
ELDOA	1	100.0%	0	0.0%	1	25.0%
YOUNG SPECIAL FORCE TRAINING	0	0.0%	0	0.0%	1	25.0%
YOUNG FIREFIGHTERS TRAINING	0	0.0%	0	0.0%	1	25.0%
KPM	0	0.0%	0	0.0%	1	25.0%
**All**	**1**	**100.0%**	**0**	**0.0%**	**4**	**100.0%**

© 2018 by the authors; licensee MDPI, Basel, Switzerland. This article is an open access article distributed under the terms and conditions of the Creative Commons by Attribution (CC-BY) license (http://creativecommons.org/licenses/by/4.0/).
